# Impact of the number of conditioning pulses on motor cortex excitability: a transcranial magnetic stimulation study

**DOI:** 10.1007/s00221-020-06010-7

**Published:** 2020-12-29

**Authors:** Petyo Nikolov, Johanna V. Zimmermann, Shady S. Hassan, Philipp Albrecht, Alfons Schnitzler, Stefan J. Groiss

**Affiliations:** 1 Institute of Clinical Neuroscience and Medical Psychology, Heinrich Heine University Düsseldorf, Düsseldorf, Germany; 2 Department of Neurology, Medical Faculty, Heinrich Heine University Düsseldorf, Düsseldorf, Germany; 3 Department of Neurology, Medical Faculty, Assiut University Hospital, Assiut, Egypt

**Keywords:** Transcranial magnetic stimulation, Facilitation, Inhibition, Conditioning stimuli

## Abstract

Conditioning transcranial magnetic stimulation (TMS) with subthreshold conditioning stimulus followed by supra-threshold test stimulus at inter-stimulus intervals (ISI) of 1–5 ms results in inhibition (SICI), while ISI at 10–15 ms results in facilitation (ICF). One concerning issue, applying ICF/SICI protocols on patients is the substantial protocol variability. Here, we hypothesized that increasing the number of CS could result in more robust ICF/SICI protocols. Twenty healthy subjects participated in the study. Motor-evoked potentials (MEP) were obtained from conditioning TMS with a varying number of conditioning stimuli in 3, 4, 10, and 15 ms ISI over the primary motor cortex. MEP amplitudes were then compared to examine excitability. TMS with 3, 5, and 7 conditioning stimuli but not with one conditioning stimulus induced ICF. Moreover, 10 ms ISI produced stronger ICF than 15 ms ISI. Significant SICI was only induced with one conditioning stimulus. Besides, 3 ms ISI resulted in stronger SICI than 4 ms ISI. Only a train of conditioning stimuli induced stable ICF and may be more advantageous than the classical paired pulse ICF paradigm.

**Supplementary Information:**

The online version contains supplementary material available at 10.1007/s00221-020-06010-7.

## Introduction

Transcranial magnetic stimulation (TMS) is an established and painless non-invasive method to study motor cortex physiology. The motor-evoked potential (MEP) amplitude can be used as marker for corticospinal excitability. Conditioning TMS is a paradigm, where the test stimulus (TS) is preceded by conditioning stimuli (CS). In this relation, several conditioning TMS techniques to study inhibitory and excitatory mechanisms have been developed. One of them is the classical paired pulse technique including short-interval intracortical inhibition (SICI) with inter-stimulus intervals (ISI) of 1–5 ms (Kujirai et al. 1993), as well as intracortical facilitation (ICF) with 10–15 ms ISI (Ilić et al. 2002). Both techniques implement one subthreshold single CS, followed by one supra-threshold TS.

ICF is thought to originate from excitatory interneurons and to be glutamate-dependent (Lazzaro et al. 1999; Ilić et al. 2002). Although ICF is considered a cortical phenomenon, additional spinal mechanisms cannot be fully excluded (Lazzaro et al. 2006). SICI, on the other hand, is viewed as pure intracortical phenomenon, which is probably based on monosynaptic inhibitory output toward the first motor neuron. It seems to be generated by low-threshold GABA-interneurons at the synaptic level (Ilić et al. 2002; Lazzaro et al. 2006).

ICF/SICI protocols have been implemented in clinical research, to determine glutamate/GABA homeostasis impairment in multiple neurological or psychiatric disorders, and already contributed to better understanding of the pathomechanisms behind some of them. However, ICF/SICI protocols can exhibit considerable outcome variability (Orth et al. 2003) and further research to increase reliability is desirable, as both techniques generally yield the potential for biomarkers (Berardelli et al. 2008; Doruk Camsari et al. 2019).

In this context, Hanajima et al. developed a protocol called triad conditioning facilitation (TCF), using three CS which was supposed to entrain the intrinsic rhythm of the motor cortex and thus strengthen the induced facilitation (Hanajima et al. 2009). Recently, we have shown that TCF could share the same mechanism of ICF, and the intensity of both CS and TS can modulate the degree of facilitation (Hassan et al. 2020). However, not only TMS intensity, but also the number of CS could modulate the degree of facilitation. Thus, in the current study, we hypothesized that increasing the number of CS could strengthen ICF.

## Materials and methods

### Participants

Twenty healthy participants (10 males; 10 females, three left-handed, mean age 24.4 ± 1.8 years) were enrolled in the study. All participants provided written informed consent in accordance with the Declaration of Helsinki (World Medical Association 2013) and the Ethical Committee of the Medical Faculty, Heinrich Heine University Düsseldorf prior to participation (Study ID: 5738R). Exclusion criteria were contraindication to TMS (e.g., due to metallic and/or magnetic implants), severe intestinal, neurological, or psychiatric diseases, the use of any medication acting on the central nervous system (e.g., benzodiazepines, anti-epileptic, and/or psychotropic drugs), blood clotting dysfunction, pregnancy, and diagnosed peripheral/retinal neuropathy.

### Transcranial magnetic stimulation

TMS was applied by a Magstim^™^ magnetic stimulator (The Magstim Co. Ltd, Whitland, UK) through a figure-of-eight coil. Eight magnetic stimulators were connected with a specially designed combining module (The Magstim Co. Ltd., Whitland, UK) to allow the application of a burst with up to eight monophasic magnetic stimuli through a single coil. The coil was placed above the primary motor cortex (M1) of the dominant hemisphere and over the individual hotspot for the first dorsal interosseus muscle (FDI). During stimulation, the coil was always positioned tangentially to the scalp with the handle pointing backwards and laterally at a 45° angle to the sagittal plane. In this way, a posterior–anterior current direction in the brain was ensured (Rothwell 1997). The configuration aims to trans-synaptically activate the corticospinal system by means of horizontal cortico-cortical connections (Lazzaro et al. 2004). After determination of the individual TMS hotspot, the active motor threshold (AMT) was defined as the lowest stimulation intensity that evoked a response of at least 100 µV during 5–10% maximal contraction of FDI in at least 5 of 10 trials using the relative frequency method (Rossini et al. 2015). While ICF was studied with 10 and 15 ms ISI (Kujirai et al. 1993), SICI was studied with 3 and 4 ms ISI (Lazzaro et al. 2006). For each ISI, the number of CS was varied between 1, 3, 5, and 7. Here, the ISI between multiple CS and between CS and TS were always the same within one condition. Single-pulse TS without CS was used as control condition. While stimulation intensity of 90% AMT was applied for CS, intensity resulting in 0.5 mV MEP response was set for TS. The order of stimulation conditions was randomized within subjects and applied in a shuffled order.

### Electromyographic recording

EMG signals were recorded from the FDI muscle with disposable Ag–AgCl surface electrodes (20 × 15 mm, Ambu, Denmark). The active electrode was placed on the muscle belly, whereas the reference was located over the base of the metacarpophalangeal joint of the index finger. EMG signals were amplified (Digitimer D360, Digitimer Ltd, Hertfordshire, UK), band passed between 10 and 5 kHz, digitized at a sampling rate of 5 kHz, and stored on a desktop computer for off-line analysis.

### Experimental design

Participants were seated in a comfortable reclining chair with arms placed on cushioned armrests during the entire experiment. Subsequently, electromyographic electrodes were attached to the FDI. The individual hot spot was determined in steps of 0.5–1 cm, starting 5 cm lateral and 1.5 cm anterior of the vertex, and defined as the spot producing the largest MEP amplitudes. The hotspot was then marked directly on the scalp with a soft-tip pen to insure constant placement of the TMS coil throughout the session. TMS intensities for AMT, as well as for the triggering of 0.5 mV MEP were determined once at the beginning and were then used throughout the experiment. To ensure subject compliance and maintain similar level of attention, MEP recordings of ICF, SICI, and single-pulse MEP were splitted into four blocks. The four blocks consisted of the following configurations:

(I) 1 CS -3 CS MEP in 10–15 ms ISI
(II) 5 CS-7 CS MEP in 10–15 ms ISI
(III) 1 CS-3 CS MEP in 3–4 ms ISI
(IV) 5 CS-7 CS MEP in 3–4 ms ISI.

Here, single-pulse MEP was measured separately in each block, which means that each block had a separate control condition. 20 MEPs were recorded for each condition (including 20 single-pulse control MEPs for each block).

Through the course of the entire experiment, muscle relaxation was monitored by an oscilloscope (Rigol DS1074B, Hirschau, Germany). Subjects were also instructed to look at a fixation cross centered in front of them and silently count the number of bursts applied to maintain similar level of attention.

### Data analysis and statistical evaluation

EMG data were analyzed with Signal Software (Cambridge Electronic Design, Cambridge, UK). Trials were visually inspected. Trials showing voluntary EMG activity immediately before the TMS pulse, as well as trials where no TMS pulse was presented due to technical reasons, were rejected from the analysis (mean = 1.3 rejected trials per condition). MEP with atypical forms and latencies were visually controlled for and not observed in our experiments, so that no MEP response with CS alone could be elicited, even with higher CS intensities (Hassan et al. 2020). Maximum peak-to-peak MEP amplitudes were determined for each trial. Subsequently, peak-to-peak MEP amplitudes were averaged over all trials of each condition. Then, MEP ratios were defined as ratio between conditioned MEP and single-pulse MEP. All MEP amplitudes were logarithmically transformed for further analysis.

Statistical evaluation was performed with SPSS. Shapiro–Wilk test was used to test for normality. Two-way repeated ANOVA was used to compare MEP ratios separately for facilitation with the factors number of CS (1, 3, 5, 7 CS) and ISI (10 and 15 ms), and for inhibition with the factors CS (1, 3, 5, 7 CS) and ISI (3 and 4 ms), respectively.

To determine the degree of MEP change of conditioning TMS compared to single-pulse TMS, absolute MEP amplitudes for each ISI were compared using one-way ANOVA (10 and 15 ms ISI) and Friedman test (3 and 4 ms ISI), respectively. For each ISI, two separate ANOVAs or Friedman tests were conducted (for 1–3 CS and 5–7 CS, respectively) in order take into account the four different blocks, since each of them had their own single-pulse control condition. If applicable, either Bonferroni or Dunn test was carried out for post hoc analysis. To confirm comparability of the single-pulse control conditions, the single-pulse MEP amplitudes for each block were compared with one-way ANOVA.

## Results

### AMT and TS intensities

Mean AMT intensity was 37% ± 2 SEM of maximal stimulator output (MPO), and mean CS intensity was 33% ± 2 SEM of MSO. Furthermore, mean TS intensity was 53% ± 3 SEM of MSO.

### Facilitation (10 and 15 ms ISI)

**Fig. 1:**
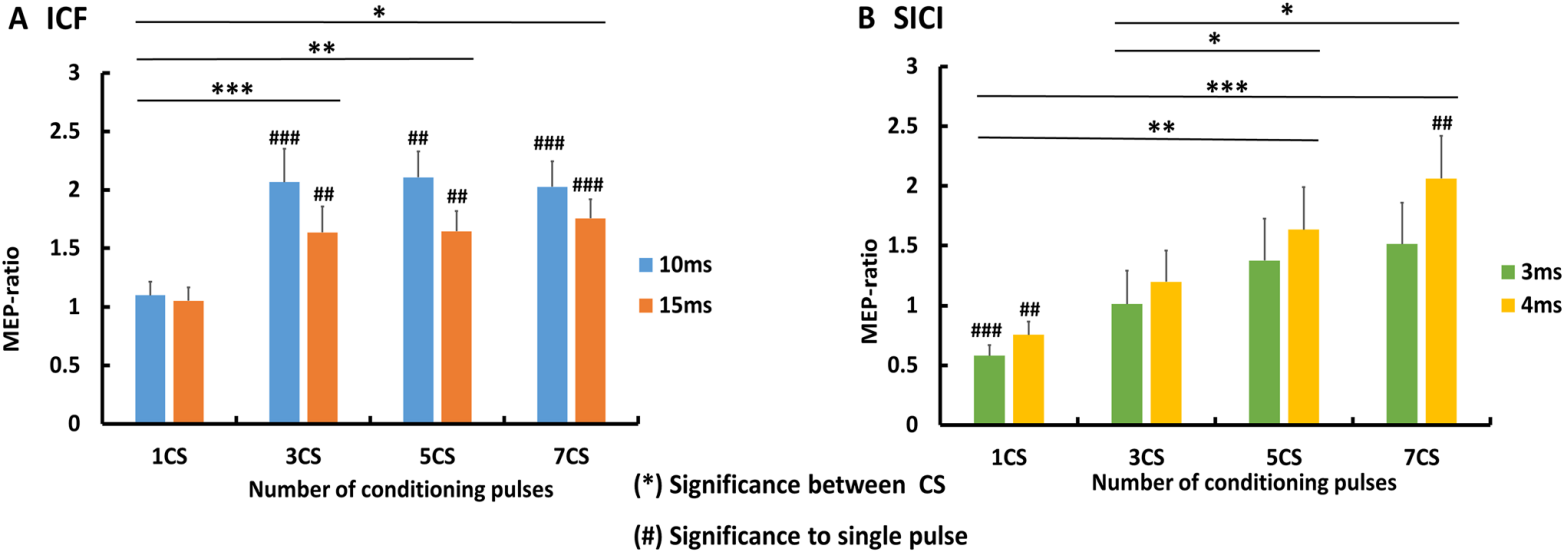
**a** Conditioning TMS with 10–15 ms (ICF); **b** conditioning TMS with 3–4 ms (SICI). Comparison between conditioning TMS–MEP ratios with variable number of conditioning stimuli. Significant differences between MEP ratios are marked with asterisk. Significant degree of change in conditioning TMS–MEP amplitudes, compared to single-pulse TMS–MEP amplitudes are marked with **#**. *p < 0.05, **p < 0.01, ***p < 0.001, #*p* < 0.05; ##*p* < 0.01, ###*p* < 0.001, error bars indicate SEM

Two-way ANOVA revealed significant main effects of CS number and ISI for MEP ratios, with no interaction between the main effects. MEP ratios significantly differed between number of CS (*F* = 10.73, *p* = 0.001). Compared to 1 CS, (mean = 1 ± 0.07 SEM), MEP ratios resulting from 3 CS (mean = 1.9 ± 0.2 SEM; *p* < 0.001), 5 CS (mean = 2.1 ± 0.2 SEM; *p* = 0.004), and 7 CS (mean = 1.9 ± 0.1 SEM; *p* = 0.014) were larger. There was no difference between 3 CS, 5 CS and 7 CS MEP ratios (*p* > 0.05 for all three), (Fig. 1a). Furthermore, MEP ratios significantly differed between ISI (*F* = 5.46; *p* = 0.031). 10 ms ISI (mean = 1.9 ± 0.1 SEM) resulted in larger MEP ratio than 15 ms ISI (mean = 1.6 ± 0.1 SEM; *p* < 0.05). For a summary of mean MEP amplitudes between conditions, please see Supplementary Table 1, Facilitation.

### Inhibition (3 and 4 ms ISI)

Two-way ANOVA revealed significant main effects of CS number and ISI over MEP ratios, with no interaction between the main effects. MEP ratios significantly differed between number of CS (*F* = 18.43, *p* < 0.001). 1 CS MEP ratio (mean = 0.7 ± 0.07 SEM) was significantly smaller than 5 CS (mean = 1.6 ± 0.2 SEM, *p* = 0.003) and 7 CS (mean = 1.9 ± 0.3 SEM, *p* < 0.001) MEP ratios. Also, 3 CS MEP ratio (mean = 1.1 ± 0.1 SEM) was significantly smaller than 5 CS (*p* = 0.014) and 7 CS (*p* = 0.004) MEP ratios. There was no difference between 1 and 3 CS MEP ratios (*p* > 0.05), as well as between 5 and 7 CS MEP ratios (*p* > 0.05) (Fig. 1b). Furthermore, MEP ratios significantly differed between ISI (*F* = 24.84, *p* < 0.001). 3 ms ISI (mean = 0.9 ± 0.1 SEM) resulted in significantly smaller MEP ratio than 4 ms ISI (mean = 1.5 ± 0.2 SEM, *p* < 0.001). For a summary of mean MEP amplitudes, please see Supplementary Table 1, Inhibition.

## Discussion

Our study has three main findings. First, a train of CS strengthens ICF, which is induced more effectively with 10 ms ISI. Second, a train with more than 3 CS do not offer additional benefit. Third, a train of CS reduces SICI, which is induced more effectively with 3 ms ISI and 1 CS.

Our results are consistent with earlier reports on the topic, where a train of 3 CS alleviated SICI with 3 ms ISI and enhanced ICF with 10 ms ISI (Calancie et al. 2018).

### Facilitation (10 and 15 ms ISI)

To our knowledge, this is the first study, where a varying number of CS was applied in an ICF protocol. We could show that a train of CS strengthens facilitation, making the ICF protocol more robust. The promoted facilitation might represent a form of short-term plasticity, shifting the interplay between facilitation and inhibition, toward facilitation. This might be attributed to temporal summation of excitatory post-synaptic potentials (EPSP) and calcium, due to the repetitive CS (Atluri and Regehr 1996; Hennig 2013). According to our results, however, more than 3 CS do not strengthen facilitation any further and do not yield extra benefit. Concerning the role of ISI in ICF protocols, facilitation had been previously described as most prominent with 10, 15, and 25 ms ISI (Lazzaro et al. 2006; Kujirai et al. 1993; Nakamura et al. 1997). Our results are consistent with the literature, as we observed significant facilitation with both 10 and 15 ms ISI. Interestingly, facilitation with 10 ms was significantly higher, compared to 15 ms ISI. An ISI of 10 ms, therefore, seems to be more robust to produce facilitation. Similarly, earlier studies have shown facilitation with an ISI of 10 ms to be more prominent (Hanajima 2009, Wessel 2019). It may also be of interest, whether ICF duration can be prolonged by the increase of CS numbers. In the present study, we did not apply ISIs longer than 15 ms. However, earlier reports using 3 CS have shown significant facilitation with 20–25 ms ISI. Whether this facilitation reflects a prolonged ICF due to EPSP summation, or underlines a different mechanism such as intrinsic rhythm of the motor cortex, is still debated (Hanajima et al. 2009; Hassan et al. 2020; Groiss et al. 2017).

Our results regarding single CS conditions did not show facilitation. This is consistent with the idea that paired pulse TMS protocols in general and the ICF protocol in particular may have a high variability (Caranzano et al. 2017; Hermsen et al. 2016; Orth et al. 2003; Wassermann 2002). However, we cannot exclude that this variability may at least partly be due to interindividual variability for optimal CS intensity to induce ICF. This might focus on the issue that a higher number of CS is more predictable in producing facilitation, than a single CS. Nevertheless, it cannot be entirely ruled out that the lack of facilitation following one CS might be due to suboptimal individual CS intensity, which may vary between individuals. Sill, given the fact that previous experiments showed that increasing CS intensity can enhance and prolong facilitation (Orth et al. 2003; Hassan et al. 2020) and at the same time too high CS intensity makes stimulation less selective and also alter the facilitation mechanism, the choice of CS intensity at 90% AMT may be regarded feasible. It is up to further research to explore facilitation variability on an individual basis while taking into account both number and intensity of CS.

### Inhibition (3–4 ms ISI)

While a train of CS strengthens facilitation, at the same time, it weakens inhibition. In fact, a train of CS might have similar impact on SICI protocols as an increase in CS intensity has. The relation between CS intensity and SICI had been well explored in the past, with SICI strength exhibiting a U-shaped function curve when varying CS intensity (Ilić et al. 2002; Kujirai et al. 1993). Indeed, the MEP amplitude change is believed to be a net outcome of inhibition and facilitation (Ni and Chen 2008), on which CS intensity has modulatory effect (Peurala et al. 2008). Analogously, a train of CS might similarly modulate the inhibition/facilitation interplay, promoting one of the two. While single CS in the range of 3–4 ms ISI causes inhibition because of the here prevailing monosynaptic inhibitory input toward the pyramidal cells, an increasing CS number probably leads EPSP summation, and thus to facilitation.

The argument above is supported by the finding that 4 ms ISI promoted facilitation with an increasing number of CS. Here, the increased number of CS might entrain EPSPs and reinforce them, as EPSPs are known to occur about 2–5 ms after initial cell depolarization (Curtis and Eccles 1959). Therefore, such summation of EPSP could explain why a train of 7 CS at 4 ms ISI resulted in significant facilitation, rather than in inhibition. Another explanation why SICI subsided after a train of CS, might be a superimposition of ICF on SICI. If a train of CS is applied at 3–4 ms ISI, then the time between the first CS and the TS would be shifted into the facilitatory ISI window around 10–20 ms.

### Outcomes significance

The reduction of variability is highly important for facilitatory TMS protocols including ICF, as this would improve protocol quality and facilitate comparability between different studies. Different strategies might be used to increase the robustness of the ICF protocol and one possibility would be to increase the CS intensity (Hassan et al. 2020). However, higher CS intensity is thought to active a larger and more heterogeneous neuronal population (Ziemann and Rothwell 2000). Instead, the present study suggests an alternative approach, by increasing the number of CS, which might limit its effects to an activation of a more uniform neuronal population. This hypothesis is supported by our findings, where MEP differed only between 1CS and a train of CS, but not between 3, 5 and 7CS. Therefore, we believe that using a train of CS might be more advantageous than increasing the CS intensity. In this way, the neurons might be targeted more selectively, without the danger of afflicting the physiological mechanism behind the protocol.

Our results could prove advantageous in establishing more robust ICF protocols, while, at the same time, reducing variability and avoiding high TMS intensities. Indeed, variability reported in ICF protocols seems to be a major issue, making ICF less reliable (Hermsen et al. 2016; Orth et al. 2003). Earlier investigation, involving ICF in Parkinson’s disease (Bologna et al. 2018; Ni and Chen 2015), dystonia (Berardelli et al. 2008), Alzheimer’s disease (Ni and Chen 2015), attention-deficit/hyperactivity disorder (Richter et al. 2007), migraine (Cosentino et al. 2018), as well as sleep research (Doeltgen and Ridding 2010), and rehabilitation medicine (Lulic et al. 2017; Singh et al. 2014) revealed either no ICF difference between control versus condition groups, or showed incongruent outcomes. Thus, ICF protocol reliability might be hampered due to technical reasons, rather than a real lack of facilitatory network difference between patients and controls. Hence, our finding might help to decrease variability when applying ICF protocols and may be useful when investigating facilitatory networks in patients with neurological diseases.

## Supplementary Information

Below is the link to the electronic supplementary material.

Supplementary file1 (PDF 425 KB)

## Data Availability

Data are available on a reasonable request from the corresponding author.

## Abbreviations

AMT: Active motor threshold
FDI: First dorsal interosseus
CS: Conditioning stimulus;
EPSP: Excitatory post-synaptic potential
ICF: Intracortical facilitation
MEP: Motor-evoked potential
MPO: Maximal stimulator output
SICI: Short-interval intracortical inhibition
STDP: Short-term dependent plasticity
TMS: Transcranial magnetic stimulation
TS: Test stimulus
