# Supplementary material for: Impact of the number of conditioning pulses on motor cortex excitability: a transcranial magnetic stimulation study
Source: Exp Brain Res. 2020 Dec 29;239(2):583–9. doi: 10.1007/s00221-020-06010-7 (PMC7936961; doi:10.1007/s00221-020-06010-7)
Supplement: Supplementary file 1 — Supplementary file1 (PDF 425 KB) [file 221_2020_6010_MOESM1_ESM.pdf]

**Facilitation (ICF)**				**Inhibition (SICI)**			
**Block I**		**Block II**		**Block III**		**Block IV**	
mean MEP (mV) ± SEM		mean MEP (mV) ± SEM		mean MEP (mV) ± SEM		mean MEP (mV) ± SEM	
**1CS, 10ms ISI**	**0.8 ± 0.1**	**5CS, 10ms ISI**	**1.4 ± 0.1 (**)**	**1CS, 3ms ISI**	**0.37 ± 0.07 (***)**	**5CS, 3ms ISI**	**0.81 ± 0.16**
1CS, 15ms ISI	0.8 ± 0.1	5CS, 15ms ISI	1.1 ± 0.1 (**)	1CS, 4ms ISI	0.51 ± 0.1 (**)	5CS, 4ms ISI	0.99 ± 0.17
**3CS, 10ms ISI**	**1.5±0.2 (***)**	**7CS, 10ms ISI**	**1.3 ± 0.1 (***)**	**3CS, 3ms ISI**	**0.65 ± 0.12**	**7CS, 3ms ISI**	**0.94 ± 0.17**
3CS, 15ms ISI	1.2 ± 0.2 (**)	7CS, 15ms ISI	1.2 ± 0.2 (***)	3CS, 4ms ISI	0.78 ± 0.12	7CS, 4ms ISI	1.29 ± 0.12 (*)
**Control MEP**	**0.8 ± 0.1**	**Control MEP**	**0.7 ± 0.07**	**Control MEP**	**0.73 ± 0.1**	**Control MEP**	**0.79 ± 0.14**

Supplementary Table1: Mean MEP amplitudes of each condition during the four experiment blocks. Please not that each block contains it separate control MEPs. Significant differences to control MEP: * $p<0.05$; ** $p<0.01$; *** $p<0.001$.
